# Egg predators improve the hatching success of salamander eggs

**DOI:** 10.1002/ece3.10427

**Published:** 2023-08-22

**Authors:** Mizuki K. Takahashi, Dominique Ruszala

**Affiliations:** ^1^ Department of Biology Bucknell University Lewisburg Pennsylvania USA; ^2^ Animal Behavior Program Bucknell University Lewisburg Pennsylvania USA

**Keywords:** *Ambystoma maculatum*, amphibians, *Lithobates sylvaticus*, oviparous animals, predator–prey interaction, spotted salamander, wood frog

## Abstract

A common challenge that oviparous animals face is securing survivorship during the vulnerable embryonic stage. One of the parental investment strategies to improve survivorship is providing physical structures to protect the embryos. In amphibians, there is a notable diversity in jelly‐layer structures surrounding eggs. Previous studies show that these jelly layers provide eggs with protection against egg predators, egg pathogens, and desiccation. However, few studies examined the cost–benefit relationship of the jelly‐layer structures. By using the predator–prey interaction between wood frog (*Lithobates sylvaticus*) tadpoles and spotted salamander (*Ambystoma maculatum*) eggs as a model system, we tested three hypotheses: (1) having the outer jelly layers would be costly to the embryos, (2) the relative benefit of the structural egg defense would become apparent and increase as the intensity of egg predation increases, and (3) a certain degree of predation would increase the hatching success of salamander embryos by mechanically thinning the thick outer jelly layers and increasing oxygen diffusion throughout an egg mass. To test these hypotheses, we conducted a factorial experiment in which we crossed four egg‐predation levels with two jelly‐layer conditions, intact or removed. We found that the jelly layers were essential in protecting spotted salamander embryos from wood frog tadpoles but that the associated cost was apparent in no‐predation treatments. The differential survivorship between intact eggs and eggs without jelly layers showed that the fitness advantage of jelly layers increased as the level of predation increased. Finally, the hatching success of intact egg masses was highest under the high predation conditions. These results imply that the evolution of the jelly‐layer thickness occurred under constant egg‐predation pressure. Given this predator–prey coevolution, egg predators may play a critical role in improving the hatching success of salamander embryos under certain conditions.

## Graphical abstract

A common challenge that oviparous animals face is securing survivorship during the vulnerable embryonic stage. One of the parental investment strategies to improve survivorship is providing physical structures to protect the embryos. We found a cost associated with the protective jelly layers surrounding the spotted salamander embryos, which egg predators mitigated.

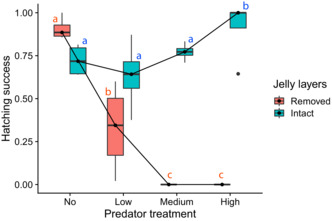

## 1 INTRODUCTION

Oviparous animals often suffer high mortality during the embryonic stage through predation, infection, and changes in abiotic factors such as temperature and moisture (Begon & Townsend, 2021; Davidson et al., 2022; Kuris, 1990; Stearns, 2000; Wilbur, 1980). In order to improve early‐life survivorship, various modes of parental investment into egg defenses have evolved across taxa, including oviposition site selection, preparation of nesting sites, and egg attendance (Mainwaring & Hartley, 2013; Mitchell et al., 2015; Okada et al., 2015; Resetarits & Wilbur, 1989; Scott, 1990; Terry et al., 2019). Parental investment can also take the form of providing physical structures protecting embryos; eggshells and extraembryonic membranes of avian and reptilian amniotic eggs serve as well‐known examples (D'Alba et al., 2021; Starck et al., 2021). Among the anamniotes, amphibian egg clutches are characterized by the diverse arrangement of jelly layers surrounding embryos (Altig & McDiarmid, 2007), providing protection against predation (Ward & Sexton, 1981), water‐mold infection (Gomez‐Mestre et al., 2006), ultraviolet‐B radiation (Licht, 2003), and desiccation (Marco & Blaustein, 1998). Jelly layers can also absorb heat, which can benefit embryo development (Licht, 1971). Whereas the benefits of those protective physical structures have been explored, relatively few studies directly examined the cost–benefit relationship associated with having such protections.

**FIGURE 1:**
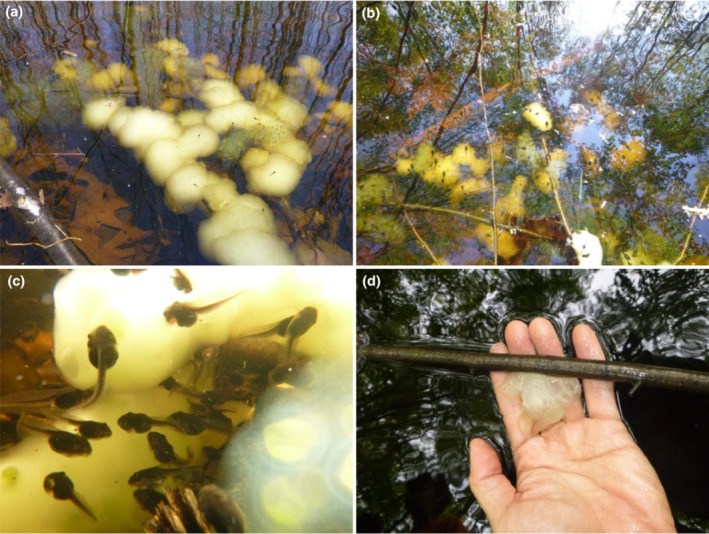
Egg masses of the spotted salamander (*Ambystoma maculatum*) observed in the field: (a) relatively early‐stage, intact egg masses surrounded by multiple jelly layers (both white and clear morphs), (b) egg masses infested with wood frog tadpoles (*Lithobates sylvaticus*), (c) wood frog tadpoles eating both jelly layers and internal embryos, and (d) a remaining egg mass after being consumed by wood frog tadpoles.

The salamanders in Family Ambystomatidae are well known for their bulky egg masses, providing excellent opportunities for cost–benefit analysis. For example, the spotted salamander (*Ambystoma maculatum*) lays egg masses surrounded by notably thick jelly layers (Figure 1a) that have likely evolved in response to egg predators such as aquatic insects and tadpoles of some anuran species (Ward & Sexton, 1981). In particular, the wood frog (*Lithobates sylvaticus*) tadpoles are voracious predators of spotted salamander eggs and can cause nearly 100% mortality when predation pressure is high (Petranka et al., 1998; Figure 1b–d). The spotted salamander and the wood frog are common pond‐breeding amphibians, and their distribution ranges largely overlap in northeastern America. Furthermore, those two species often breed in the same vernal pools, and wood frogs typically breed before spotted salamanders (Andrews & Talmage, 2021), resulting in the predator–prey interaction where tadpoles eat salamander eggs.

Wood frog tadpoles are omnivorous and forage plant materials such as algae and dead leaves. They also consume animal materials, including dead animal tissues and live eggs of other pond‐breeding amphibians (Altig et al., 2007; Petranka et al., 1998; Schiesari et al., 2009). Even when there are abundant plant materials, wood frog tadpoles seek to forage animal tissues (Figure 1b–d); those that consume additional animal‐based protein can grow larger and develop faster than those fed only plant materials (Chuirazzi et al., 2021). The fitness advantage of consuming animal materials explains tadpoles' high foraging pressure on salamander eggs and the consequent anti‐predatory egg defense by the spotted salamander in the form of thick protective jelly layers.

Despite the apparent benefit, the outer jelly layers challenge spotted salamander embryos by interfering with oxygen diffusion throughout the egg mass (Pinder & Friet, 1994). In fact, providing sufficient oxygen for embryos is a common challenge among vertebrates that lay eggs in aquatic habitats. In species such as fish and aquatic salamanders that provide parental care, parental individuals, often males, supply embryos with more oxygenated water by fanning with tails and fins (only fish; Gross & Sargent, 1985; Jones & Reynolds, 1999; Okada et al., 2015), which improves hatching success (Hale et al., 2003; Kobara, 1985). In some fish species, a parental male cannibalizes a portion of the clutch in response to low oxygen levels, resulting in the increased hatching success of the remaining embryos (Klug et al., 2006; Payne et al., 2002).

In pond‐breeding amphibians without parental care, passive diffusion is not enough to provide sufficient oxygen for embryos, and photosynthetic symbiotic algae associated with the jelly layers play an essential role in supplying additional oxygen (Pinder & Friet, 1994). In a lab experiment, spotted salamander embryos surrounded by the intact jelly layers suffered nearly 100% mortality under a constantly dark condition, whereas embryos without the outer jelly layers under the same dark condition achieved ~60%–80% hatching success, which was comparable to the hatching success of the intact egg mass under a 12 h light and 12 h dark cycle (Hale et al., 2017). The data from the field also suggest that embryos in intact masses can suffer significant mortality, especially in ponds with hypoxic conditions (e.g., high canopy coverage; Sacerdote & King, 2009). These lines of evidence led to the hypotheses that (1) having the outer jelly layers would be costly to the embryos even under normal light conditions, and (2) the relative benefit of the structural egg defense would become apparent and increase as the intensity of egg predation increases. Furthermore, the study introduced above (Hale et al., 2017) implies an intriguing possibility that (3) a certain degree of predation would increase the hatching success of salamander embryos by mechanically thinning the thick outer jelly layers and increasing oxygen diffusion throughout an egg mass. To test these hypotheses, we conducted a laboratory experiment in which we reared spotted salamander clutches with and without the protective jelly layers under four different levels of tadpole predation.

## 2 MATERIALS AND METHODS

### 2.1 Ethics statement

The procedures and husbandry used in this study were approved by Bucknell University IACUC (Institutional Animal Care and Use Committee). We conducted sample collection under the scientific collection permit issued by the Pennsylvania Fish and Boat Commission.

### 2.2 Field collection

The spotted salamander (*A. maculatum*) and the wood frog (*L. sylvaticus*) are common pond‐breeding amphibians throughout their distribution ranges in northeastern America. Our field collection site (Union County, Pennsylvania, USA) is located within the core region of the distribution ranges. During the spring of 2015, we monitored several forested vernal ponds daily. We collected the freshly laid egg masses of spotted salamanders on April 4 after the major breeding event a day before, ensuring that all egg masses were the same age. Egg masses of spotted salamanders come in two morphs, white and clear, and we used white egg masses because the white morph is dominant in this region. The collected egg masses were individually housed in 7.2 L rectangle plastic containers half‐filled with pond water (container size: 28 cm [L] × 15 cm [W] × 11 cm [H]) under 10°C and a 12 h light and 12 h dark cycle. After we processed the salamander egg masses and prepared them for the experiment, we collected wood frog tadpoles from the same vernal pond on April 24.

### 2.3 Experimental design

We used 32 white egg masses for the experiment. We randomly assigned them to eight treatments based on a full factorial design of two independent variables: four levels of predation treatment (zero, low, medium, or high predation), and two levels of jelly‐layer manipulation (intact or removed). We replicated each of the six treatments four times, resulting in 32 experimental units. We blotted each egg mass with paper towels and weighed each (*n* = 32, mean ± SE = 268.1 ± 13.0 g). We then gently removed jelly layers by hand from half of the egg masses and recorded the number of embryos of each (*n* = 16, mean ± SE = 84.6 ± 8.5). We estimated the number of embryos of the remaining intact egg masses based on the linear regression formula between the number of embryos and the weight of the egg mass from those without jelly layers (*p* < .0001; *y* = 0.4071*x* + 22.851, *R* ^2^ = 0.6753; Petranka et al., 1998). We placed each egg clutch into a 7.2 L rectangle plastic container half‐filled with aged tap water (container size: 28 cm [L] × 15 cm [W] × 11 cm [H]) and randomly arranged them in a growth chamber set at 10°C with a 12 h light and 12 h dark cycle.

On April 25, after confirming that exposed embryos were developing normally, we began the experiment by adding wood frog tadpoles to 16 containers pre‐assigned for the predation treatments. We calculated the average number of spotted salamander embryos per egg mass (mean ± SE = 86.3 ± 5.8 eggs for all 32 egg masses) and set the number of tadpoles in each predator treatment as 0 tadpoles (no predation), 50 tadpoles (low predation), 100 tadpoles (medium predation), or 200 tadpoles (high predation). We then adjusted the actual number of tadpoles added to each container based on the number of embryos in a given container. The range of the tadpole number varied from 0 to 272 per container with an area of 710 cm^2^, which was well within the naturally observed range of the tadpole density (Takahashi per. Obs.).

Tadpoles in a natural pond have unlimited access to leaf litter as food. Thus, we fed tadpoles weekly with rabbit food ad‐lib. We also cleaned off excess food and excrement and performed a 50% water change of all containers weekly. The temperature in the growth chamber was kept at 10°C until May 18th and then was raised to 15°C to simulate a rapidly rising spring temperature in the field. We monitored all containers daily and recorded any hatchlings to calculate hatching success (the number of hatchlings divided by the number of embryos) and embryonic period per container. Developmental stages and sizes of hatchlings were not recorded because hatchlings were used for another experiment, and taking precise measurements of these traits would have required euthanization. We terminated the experiment on June 8 when all embryos had hatched out or been consumed by tadpoles. In one container assigned for high predation with jelly layers, the actual number of hatchlings was greater than the estimated number of embryos based on the linear regression formula. Thus, we corrected the survivorship of this egg clutch to 1.

### 2.4 Statistical analysis

We conducted all statistical analyses using the software R 4.0.5 (R Core Team, 2021). Because the hatching success data was based on a continuous probability distribution in the unit interval (0,1) including 0 and 1, we first transformed the data by using the formula [(*y* · (*n* − 1) + 0.5)/*n*] where n is the sample size (Smithson & Verkuilen, 2006). We then conducted beta regression using the “betareg” package (Cribari‐Neto & Zeileis, 2010) to analyze the effects of the predation treatment, the jelly‐layers treatment, and their interaction on the hatching success of salamander embryos. Prior to the beta regression, we also confirmed that the AIC score of the beta distribution model was improved (delta AIC = 38.4) compared to that of the Gaussian distribution model by using the “MASS” (Venables & Ripley, 2002) and the “fitdistrplus” package (Delignette‐Muller & Dutang, 2015). We analyzed the embryonic period data using a generalized linear model with Gaussian error distribution after confirming the normality and the homogeneity of the data. For all post‐hoc pairwise comparisons, we conducted Tukey's honestly significant difference (HSD) tests using the “emmeans” package (Lenth, 2021).

## 3 RESULTS

### 3.1 Hatching success

**FIGURE 2:**
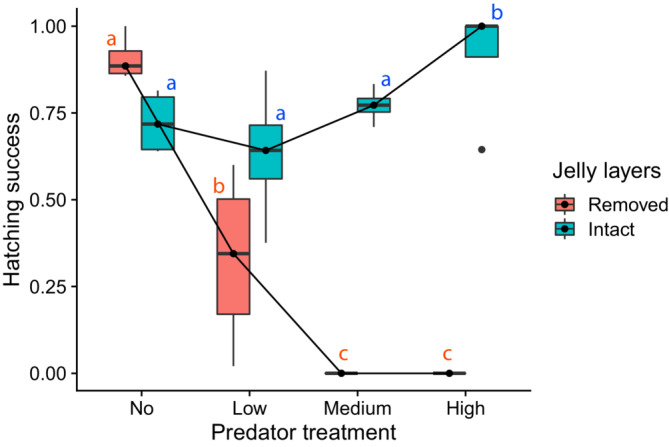
Boxplots showing the hatching success of spotted salamander embryos (*Ambystoma maculatum*) under eight different treatments in a lab experiment with two independent variables fully crossed: four predation levels (no, low, medium, and high) and two jelly‐layer levels (jelly layers removed or intact). Predation treatments consisted of varied numbers of wood frog tadpoles (*Lithobates sylvaticus*). Spotted salamander embryos are surrounded by thick jelly layers, which were gently removed to create “removed jelly‐layer” treatments. There was a significant interactive effect between the two independent variables. Within the removed jelly‐layer group, hatching success was highest under no predation followed by that under low predation, while no embryos successfully emerged under medium and high predation treatments (statistical differences indicated by red alphabets). Within the intact jelly‐layer group, hatching success under high predation was greater than those of any other predation treatments (statistical differences indicated by blue alphabets).

In total, 1401 hatchlings successfully emerged from 32 egg clutches (range: 0–125; mean ± SE = 43.8 ± 6.0 hatchlings). Overall hatching success in the absence of predations (no predation: 81.5% ± 4.4) was similar to the values reported from a previous lab experiment (Hale et al., 2017). Hatching success was influenced by both predation treatment and jelly‐layer manipulation (Figure 2; predators: *χ* ^2^ = 317.4, *p* < .001; jelly‐layer manipulation: *χ* ^2^ = 138.9, *p* < .001). Hatching success under no predation treatment was greater than the rest of the predation treatments (no predation: 81.5% ± 4.4; low predation: 48.0% ± 9.6; medium predation: 38.6% ± 14.6; high predation: 45.6% ± 17.7) and intact egg masses overall resulted in better hatching success than egg without jelly‐layers (intact: 76.0% ± 4.2; removed: 30.9% ± 10.0). Furthermore, there was a strong interactive effect between the predation and jelly‐layer manipulation (*χ* ^2^ = 367.6, *p* < .001). Post‐hoc tests revealed that the hatching success of egg clutches without jelly layers was highest under no predator treatment, followed by that under low predator treatment, while no embryos without jelly layers successfully emerged under medium or high predator treatment (Figure 2; post‐hoc tests, 90.7% ± 0.03 in no predation >32.8% ± 0.13 in low predation [*p* < .001] > 0% in medium predation [*p* = .005] = 0% in high predation [*p* = 1.000]). In contrast, intact egg masses achieved the highest hatching success under high predation (Figure 2; post‐hoc tests, 91.1% ± 0.09 in high predation >77.2% ± 0.025 in medium predation [*p* = .043], high predation >63.3% ± 0.10 in low predation [*p* = .001], high predation >72.3% ± 0.046 in no predation [*p* = .012]). There were no significant differences in the hatching success of intact egg masses among no, low, and medium predator treatments.

**FIGURE 3:**
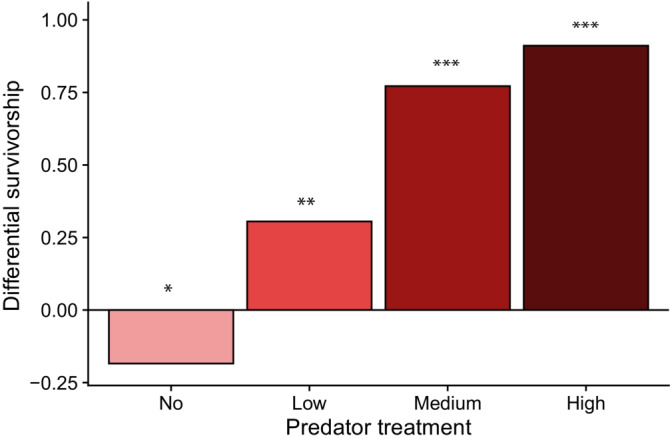
Differential survivorship between intact eggs and eggs without jelly layers of spotted salamanders (*Ambystoma maculatum*) under four different predation treatments. Differential survivorship is defined as (hatching success of intact eggs)—(hatching success of eggs without jelly layers). Asterisks indicate statistical significance in differential survivorship between intact and removed egg masses: *p < .05, **p < .001, and ***p < .0001.

In the absence of predation, hatching success was significantly greater in eggs without jelly layers than intact eggs (*p* = .021). In low, medium, and high predation, hatching success was significantly greater in intact eggs (post‐hoc test, *p* = .003, *p* < .001, and *p* < .001, respectively). As a result, the differential survivorship, which was defined as (hatching success of intact egg mass) − (hatching success of egg mass without jelly layers), increased as the predation intensity increased (Figure 3).

### 3.2 Embryonic period

**FIGURE 4:**
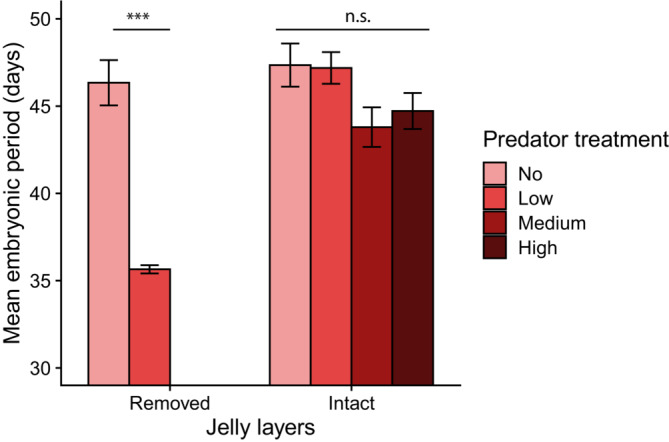
Effects of predator (wood frog tadpoles, *Lithobates sylvaticus*), the presence of jelly‐layers surrounding salamander embryos, and an interaction between the two independent variables on the embryonic period (mean ± SE) of spotted salamanders (*Ambystoma maculatum*). No data is shown under medium and high predation treatments within the removed jelly‐layer group because no embryos successfully hatched under those conditions. There was a significant interactive effect between the two independent variables. Within the removed jelly‐layer group, predatory tadpoles induced significantly earlier hatching (***p < .0001), whereas within the intact jelly‐layer group, there were no significant differences in embryonic period among the predation treatments (n.s.: non‐significant difference).

Because there was no successful hatching from egg clutches without jelly layers under medium and high predation treatments, there was no embryonic period data from those treatments. Even without the data from those treatments, we found significant effects of predation treatment and jelly‐layer manipulation as well as the interactive effect between the two main effects (Figure 4; predation treatment: *χ* ^2^ = 36.3, *p* < .0001; jelly‐layer manipulation: *χ* ^2^ = 36.6, *p* < .001; interaction: *χ* ^2^ = 25.8, *p* < .001). Although there was a trend of earlier hatching from intact egg masses under greater predation pressure (Figure 4), there were no statistically significant differences in the embryonic period among the predator treatments. When jelly layers were removed, however, low predation treatment induced significantly earlier hatching than no predation treatment (35.7 days ±0.2 in low predation vs. 46.3 days ±1.3 in no predation, post‐hoc test *p* < .001).

## 4 DISCUSSION

Our results showed that the jelly layers were essential in protecting spotted salamander embryos from wood frog tadpoles (Figure 2), corroborating the findings of the previous study in which tadpoles of the Southern Leopard Frog (*Lithobates sphenocephalus* or *Rana sphenocephala*) were used as predators along with other insect predators (Ward & Sexton, 1981). With the jelly layers removed, all eggs were entirely consumed by tadpoles in mid‐ and high‐predation treatments. However, the fitness advantage of having protective jelly layers was apparent only when predators were present. In fact, in the absence of predators the hatching success of intact egg masses (72.3% ± 0.046) was lower than that of eggs without jelly layers (90.7% ± 0.03; Figures 2 and 3), supporting the first hypothesis about the cost of being surrounded by thick jelly layers even under normal light conditions. The differential survivorship between intact eggs and eggs without jelly layers showed that the fitness advantage of jelly layers increased as the level of predation increased (Figure 3), supporting the second hypothesis. Finally, our third hypothesis was that the cost of physical egg protection could be mitigated by egg predators that mechanically remove the surrounding jelly layers. The results showed that the high predation pressure improved the hatching success of intact egg masses (91.1% ± 0.09; Figure 2), supporting the hypothesis.

A major factor contributing to the cost associated with the jelly layers was likely to be the hindrance of oxygen diffusion among the embryos (Kerney, 2011; Pinder & Friet, 1994). This cost becomes significant when insufficient light is provided for endosymbiotic algal photosynthesis to provide embryos oxygen (Hale et al., 2017) and when the level of dissolved oxygen is low (Sacerdote & King, 2009). Hale et al. (2017) found nearly 100% mortality of intact eggs of the spotted salamander under constant dark conditions. However, when jelly layers were removed, light conditions did not affect hatching success (60%–80%), hatching mass, age at hatching, and developmental stage at the hatching of eggs. Sacerdote and King (2009) monitored the hatching success of spotted salamander eggs in eight ponds in the field over 3 years by individually securing egg masses in mesh enclosures. Those authors repeatedly found high mortality in the ponds with low levels of dissolved oxygen (average hatching success = 32.9% ± 0.05, range = 0–100%, *N* = 56). Building onto these studies showing the cost of the protective jelly layers under unfavorable conditions, our study shows that the cost still exists under normal light conditions. In addition to the cost associated with oxygen diffusion, we observed dead embryos often positioned in the center of the egg mass, and they appeared developmentally ready to hatch. This may suggest greater oxygen demand as embryos develop more, resulting in mortality later in the embryonic developmental stages. It is also possible that thick jelly layers physically challenge the hatching of embryos. Overall, evidence from the previous and present studies suggests that the cost of having thick protective layers can impose tangible adverse effects on the hatching success of spotted salamander embryos under the range of naturally occurring conditions.

Interestingly, egg predators that mechanically thinned the surrounding jelly layers mitigated the cost of physical egg protection. The hatching success of intact egg masses under high predation treatment was similar to that of removed egg masses in no predation treatment (91.1% ± 0.09 vs. 90.7% ± 0.03, Tukey's HSD, *p* = .9912); these two treatments achieved the highest hatching success among all eight treatment groups (Figure 2). Applying these findings to the field conditions, egg predators may play a critical role in improving hatching success of salamander embryos, particularly under conditions where light or dissolved oxygen is limited. However, high tadpole density can also cause nearly 100% mortality among spotted salamander eggs in the field (Petranka et al., 1998; Figure 1b–d), which our predation treatments did not capture. Thus, hatching success depends on the intricate balance between egg predation level and abiotic factors.

Environmentally cued hatching is widespread across various animal groups, including amphibians (Warkentin, 2011). In general, egg predators induce earlier hatching, and larval predators delay hatching in amphibians (Warkentin, 2011). These plastic responses are considered adaptive because early‐stage amphibians increase their chance of survival by hatching earlier to escape egg predator's attack or by hatching later with more developed swimming performance to escape larval predators. However, Anderson and Petranka (2003) found that odonate larval predators did not affect the hatching timing of the spotted salamander embryos. Our results also showed that the embryonic period of the intact eggs did not change depending on the levels of egg predation (Figure 4). When jelly layers were removed, however, those embryos exposed to the low level of predation hatched significantly earlier (by ~10 days) than those without predation. Alternatively, tadpoles selectively consumed slow‐developing embryos, resulting in the overall shorter embryonic period. However, this is unlikely to explain the result because, in removed egg masses, no embryos under no predation treatments hatched as early as those under low predation. In addition, Gomez‐Mestre et al. (2006) found that egg pathogen (water molds) accelerated the hatching of the spotted salamander embryos only when the jelly layers were removed. These differences in response arose presumably because, without the outer jelly layers, the embryos perceived the chemical cues of egg predators/pathogens to a greater degree (Ireland et al., 2007; Sih & Moore, 1993) and also because tadpole predators physically disturbed the embryos to stimulate hatching (Jung et al., 2022; Warkentin, 1995, 2000).

Most animals are oviparous, and their common challenge is ensuring survivorship during the vulnerable embryonic stages (Stearns, 2000; Wilbur, 1980). Amphibians, in particular, come with notably diverse arrangements of jelly‐layer structures surrounding eggs, which reflect adaptation to biotic and abiotic variables as well as phylogenetic constraints (Altig & McDiarmid, 2007; Salthe, 1963). Although the selective factors involved are still being determined in many taxa, the level of egg predation, gas exchange, and the physical challenge of hatching through jelly layers are likely among the most vital factors imposing stabilizing selection on the thickness of the jelly layers for pond‐breeding amphibians. For example, the Jefferson salamander (*A. jeffersonianum*) and the Marbled salamander (*A. opacum*) are two congenic pond‐breeding salamanders which are commonly found with the spotted salamander and the wood frog throughout much of their distribution ranges. The Jefferson salamander breeds earlier in the spring than the spotted salamander and other pond‐breeding frogs. By the time tadpoles become large enough to forage the spotted salamander egg masses, the majority of the Jefferson salamander eggs have already hatched. The thickness of the Jefferson salamander egg masses is notably thinner than those of the spotted salamander. The Marbled salamander breeds in the fall on land immediately adjacent to a pond, the egg clutch is then guarded by the female, and the eggs hatch in late fall when they are flooded with pond water. In this species, the risk of egg predation is presumably much lower, and no outer jelly layers surround individual egg capsules. In this way, the family Ambystomadae, containing 32 species with vastly different jelly‐layer arrangements (Petranka, 1998), is an excellent model to further investigate the selective factors shaping diverse egg morphology, which include not only various egg predators (Ward & Sexton, 1981; this study) but also pathogens (Gomez‐Mestre et al., 2006), oxygen demand (Pinder & Friet, 1994), ultraviolet‐B radiation (Licht, 2003), desiccation (Marco & Blaustein, 1998), and oviposition timing (Andrews & Talmage, 2021).

Amphibians and other oviparous animals have co‐evolved with egg predators, and selection is predicted to favor the properties of physical egg protection that maximize the overall fitness of the egg clutch (Stearns, 1989, 2000). Our results suggest that (1) there is a trade‐off of having physical egg‐defense structures, (2) the cost–benefit relationship of physical egg defense changes depending on predation level, and (3) certain intensities of egg predation can improve hatching success, likely resulting from the evolution of the jelly‐layer thickness under constant egg‐predation pressure. The broader applicability of these findings and whether females are able to adjust the egg defense level plastically are yet to be determined.

## AUTHOR CONTRIBUTIONS

**Mizuki K. Takahashi:** Conceptualization (lead); data curation (supporting); formal analysis (lead); investigation (lead); methodology (equal); project administration (lead); visualization (lead); writing – original draft (lead); writing – review and editing (supporting). **Dominique Ruszala:** Conceptualization (supporting); data curation (lead); formal analysis (supporting); investigation (lead); methodology (equal); project administration (supporting); visualization (supporting); writing – original draft (supporting); writing – review and editing (lead).

## FUNDING INFORMATION

None.

## CONFLICT OF INTEREST STATEMENT

None.

## Data Availability

Hatching and incubation‐period data are available via: https://doi.org/10.5061/dryad.bnzs7h4gn.
